# High consistency of trophic niches in generalist arthropod species (Oribatida, Acari) across soil depth and forest type

**DOI:** 10.1002/ece3.9572

**Published:** 2022-12-12

**Authors:** Jing‐Zhong Lu, Peter Hans Cordes, Mark Maraun, Stefan Scheu

**Affiliations:** ^1^ Johann‐Friedrich‐Blumenbach Institute of Zoology and Anthropology University of Göttingen Göttingen Germany; ^2^ Center of Biodiversity and Sustainable Land Use University of Göttingen Göttingen Germany

**Keywords:** Douglas fir, functional trait, soil fauna, species coexistence, trophic plasticity

## Abstract

Many traits including trophic niche parameters are attributed to species. However, generalist species may vary in trophic niches with environments, making species‐based knowledge hard to extrapolate beyond local food webs. Here we tested trophic consistency in oribatid mite species (Acari), one of the most abundant arthropods that occupy all trophic levels in soil food webs. We used stable isotope analysis to compare trophic niches of 40 Oribatida species that co‐occur in litter (O_L_) and soil (0–5 cm, mainly O_F/H_, A_H_) of five forest types (native European beech, non‐native Douglas fir, range‐expanding Norway spruce, two beech–conifer mixed forests). Although stable isotope signatures of bulk material differed between litter and soil, ^13^C and ^15^ N values of Oribatida species were remarkably stable irrespective of soil depth. Furthermore, Oribatida were more enriched in ^13^C in European beech than in coniferous forests, but forest type little affected ^15^ N values of Oribatida across a range of site conditions. We conclude that Oribatida species occupy virtually identical trophic niches (δ^13^C and δ^15^N values) irrespective of the soil depth they colonize and that forest management including non‐native tree species little affects trophic position (δ^15^N values) of oribatid mites. Our findings suggest that the trophic position can be used as a trait in community analysis of Oribatida across forest ecosystems. Our results further indicate that trophic niches of generalist species can be highly consistent irrespective of environment.

## Graphical abstract

We studied variations in trophic niches with soil depth and forest types in 40 oribatid mite species using stable isotopes. Trophic positions were remarkable constant irrespective of soil depth and forest type. Therefore, trophic position may be used as quantitative trait in oribatid mite species across different environments.

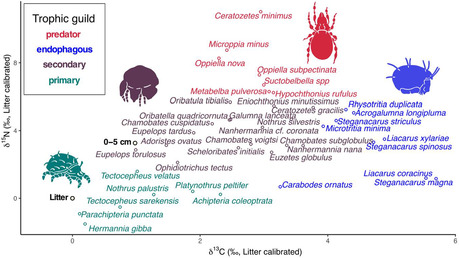

## 1 INTRODUCTION

Generalist species use a wide range of resources and may vary in trophic niches with environments (Büchi & Vuilleumier, 2014; Colborn et al., 2020; Krause et al., 2019). Trophic niche is an essential concept in ecology that describes the resource utilization of organisms (Holt, 2009; Schoener, 2009). However, many trophic niche parameters are attributed to species, without knowing how they change with environmental settings (Matthews et al., 2010; Potapov et al., 2021), posing a challenge to extrapolate trophic niches of species beyond local communities (Moretti et al., 2017; Violle et al., 2007). This particularly applies to soil detritivorous animals which have been assumed to predominantly live as generalists feeding on a wide range of resources (Digel et al., 2014; Scheu & Setälä, 2002). On the other hand, trophic niche differentiation has been detected in major groups of soil microarthropods, including oribatid mites, collembolans, and mesostigmatid mites (Chahartaghi et al., 2005; Klarner et al., 2013; Schneider et al., 2004). Niche differentiation contributes to the coexistence of species, but high variation in trophic niches of individuals will cause niche overlap among species hampering species coexistence. Overall, it remains poorly understood how trophic niches of soil microarthropods vary among microhabitats and environments, such as soil depth and forest type.

Microarthropods dominate in the uppermost horizons of forest soils, especially the organic horizons (Arribas et al., 2021; Mitchell, 1978; Pande & Berthet, 1975). Stable isotope values of carbon (^13^C) and nitrogen (^15^ N) are typically enriched with soil depth (Högberg et al., 2020). Therefore, the same microarthropod species from different depths may be associated with changes in stable isotope values parallel to that of litter and soil (Högberg et al., 2020; Potapov et al., 2019). Further, in organic matter at more advanced stages of decay, the proportion of bacteria and mycorrhizal fungi increases relative to saprotrophic fungi, which dominate at earlier stages of decay in the litter layer (Lindahl et al., 2007; Lu & Scheu, 2021). Soil depth provide a small‐scale vertical environmental gradient for studying trophic–environmental relationships, but trophic consistency between soil depths has not been rigorously tested (but see Scheu & Falca, 2000).

Similar to depth, forest types vary in litter quality and microbial communities (Albers et al., 2004; Lu & Scheu, 2021). Non‐native tree species may provide limited resources for associated biota, as indicated by lower microbial biomass and changes in microbial community structure (Lu & Scheu, 2021). In central Europe, non‐native Douglas fir is an interesting tree species for forestry due to its timber quality and resistance to climate change, but little is known about the effects of Douglas fir on the trophic structure of soil food webs compared to native European beech forests (Hobbie et al., 2007; Kriegel et al., 2021; Schmid et al., 2014). Litter decomposition and nutrient cycling are generally faster in deciduous than coniferous forests (Albers et al., 2004; Berger & Berger, 2012). Faster decay of litter by microorganisms drives changes in bacteria and fungi channels, leading to variations in depth gradients of stable isotope signatures and of food resources among forests (Clemmensen et al., 2013; Lu & Scheu, 2021). Different basal resources can shift food web structure (Nagelkerken et al., 2020; Scheu & Falca, 2000; Susanti et al., 2021), but to what extent forest types affect trophic niches of soil microarthropod species has not been comprehensively quantified.

Oribatida mites (Oribatida, Acari) are among the most abundant soil microarthropods and occupy all trophic levels in soil food webs (Maraun et al., 2011; Schaefer, 1990). Oribatida species have been proposed to occupy similar trophic niches irrespective of depth, forest type or nitrogen deposition (Gan et al., 2014; Scheu & Falca, 2000; Schneider et al., 2004). By contrast, laboratory feeding experiments suggest that soil microarthropods are food generalists (Bokhorst et al., 2007; Buse & Filser, 2014; Maraun et al., 1998), and recent studies further indicate that resource availability may shift basal resources and trophic positions of Oribatida species under different land use (Krause et al., 2019; Maraun et al., 2020). The contrasting response calls for more analyses including a wide range of Oribatida species. Including feeding guilds of oribatid mites better represents their multitrophic biodiversity and may allow deeper insight into the interrelationship between environments and guild‐specific trophic ecology of Oribatida species.

Here, we tested the trophic consistency of 40 Oribatida species in litter and soil of five forest types using bulk stable isotope analysis of ^13^C and ^15^ N. Stable isotope ratios of ^13^C/^12^C suggest the use of different basal resources, and those of ^15^ N/^14^ N ratios indicate the trophic position of consumers in soil food webs (Potapov et al., 2019; Tiunov, 2007). The forest types were selected to represent major current and future forest stands in Central Europe, including pure stands of European beech, Norway spruce, Douglas fir, and the two conifer–beech mixtures. Forest types were replicated covering a range of water and soil conditions (Foltran et al., 2020; Lwila et al., 2021). We hypothesized that (1) stable isotope ratios of Oribatida species differ between litter and soil, and (2) the variation of stable isotope ratios in Oribatida species with environments depends on their feeding guilds. Further, we hypothesized that (3) trophic niches of Oribatida species differ most between European beech and Douglas fir forests.

## 2 METHODS

### 2.1 Study sites

The study was conducted in 40 forest stands located in Northern Germany (4 sites x 2 site conditions x 5 forest types). The sites covered a wide range of soil and water conditions (Figure S1). Four southern sites stocked on Cambisol and Luvisol, with mean annual precipitation of 821–1029 mm. Four more northern sites were located on nutrient‐poor out‐washed sand, with soil type Podzol and mean annual precipitation of 672–746 mm. Hereafter, we refer to the southern sites as loamy and to the northern sites as sandy. Each site comprised three pure stands of European beech (*Fagus sylvatica* L.), Norway spruce (*Picea abies* [L.] Karst.), and Douglas fir (*Pseudotsuga menziesii* (Mirbel) Franco.) as well as two beech–conifer mixtures (European beech/Douglas fir and European beech/Norway spruce). Focal tree species in pure stands on average comprised more than 90% of total basal area, while in the two mixed stands focal tree species on average accounted for 34% for European beech and 58% Douglas fir, and for 56% European beech and 37% Norway spruce. Trees were on average more than 50 years old. More details about the sites are given in Ammer et al. (2020), Foltran et al. (2020), and Lu and Scheu (2021).

### 2.2 Field sampling

Microarthropods were sampled by using a soil corer (ø 20 cm) between November 2017 and January 2018. One soil core was taken in each of the 40 forest stands with the samples taken between trees of the same (pure stands) or different species (mixed stands). Samples were separated into litter (O_L_) and underlying 5 cm soil depth (mainly O_F/H_, A_H_), hereafter referred to litter and soil representing different soil depths. Soil arthropods were extracted separately for litter and soil using high‐gradient heat extraction (Kempson et al., 1963). Animals were collected in 50% diethylene glycol and then transferred into 70% ethanol. Storage in ethanol can change the isotope composition of soil microarthropods, but the changes are small compared to the difference between species (Fábián, 1998; Kudrin et al., 2015; Sticht et al., 2006). Species were identified using the key of Weigmann (2006). Separate soil samples (ø 5 cm) were taken in close vicinity to the 20 cm soil core and were separated into litter and underlying 5 cm soil depth for bulk stable isotope analysis (Lu & Scheu, 2021).

### 2.3 Species selection

We selected species that occurred both in litter and soil from the same soil core, as our primary focus was to understand the variation in isotope values of species with soil depth. According to published data and expert evaluation, species were ascribed a priori to four feeding guilds including primary decomposers, secondary decomposers, endophagous species (incorporating CaCO_3_ into their cuticle), and predators/scavengers (Maraun et al., 2011; Maraun et al., 2022; Schneider et al., 2004). From each soil core, the same species were selected from each litter and soil, and two to three Oribatida species (adults) of different feeding guilds were selected for stable isotope analysis. This resulted in 188 populations covering 40 Oribatida species. Females were used in two species with sexual dimorphism, that is, *Acrogalumna longipluma* and *Adoristes ovatus* (Weigmann, 2006). Since the community composition of Oribatida differs between forest types resulting in limited overlap of species among forest types (Lu, 2021), 27 of the selected 40 species occurred in more than one forest type (Table S1). We applied linear‐mixed effects modeling to control for the difference in species among forest types, allowing to inspect forest type effects on trophic niches of Oribatida species (see Section 2.5: data analysis).

### 2.4 Stable isotope measurements

We used bulk stable isotopes (^13^C/^12^C and ^15^ N/^14^ N ratios) of Oribatida species to quantify their trophic niches. Stable isotope values of litter and soil were measured and used as baseline for comparing Oribatida species across forest types (Klarner et al., 2013; Potapov et al., 2019). Different numbers of individuals were used for stable isotope analysis depending on body size of the species; the weight of each sample ranged from 6.5 μg in *Ophidiotrichus tectus* to 241 μg in *Steganacarus magnus* (Table S2); the same number of individuals was used for each Oribatida species from different depths of the same soil core. Litter and soil were dried at 60°C for 48 h, and ground using a ball mill. After weighing into tin capsules, the natural abundance of stable isotope ratios of carbon (^13^C/^12^C) and nitrogen (^15^ N/^14^ N) of bulk litter and soil was determined by a coupled system of an elemental analyzer (NA 1110, CE‐instruments, Rodano) and a mass spectrometer (Delta Plus, Finnigan MAT). Oribatid mites were transferred to tin capsules and dried at 60°C for 48 h and isotopic signatures were analyzed by a second coupled system of an elemental analyzer (Flash 2000, Thermo Fisher Scientific) and a mass spectrometer (Delta V Advantage, Thermo Electron). Animal samples with dry weight < 100 μg were analyzed using a near‐conventional setup developed for small sample amount; amounts of 0.6 μg N animal tissue were sufficient for accurate isotopic estimation with <1‰ standard error (Langel & Dyckmans, 2014). Atmospheric nitrogen and Vienna PeeDee belemnite were used as primary standards. Acetanilide (C_8_H_9_NO, Merck) was used as internal working standard. Natural variation in stable isotope ratios of carbon and nitrogen (δX) was expressed as δ*X* (‰) = (*R*_sample_−*R*_standard_)/*R*_standard_ х 1000, with R being the ratio between the heavy and light isotopes (^13^C/^12^C or ^15^ N/^14^ N).

### 2.5 Data analysis

To confirm the feeding guilds that were assigned a priori, we measured stable isotope values of the Oribatida species included in this study and calibrated them to litter from the same forest stand where they had been sampled (Klarner et al., 2013; Potapov et al., 2019; Table S1). Overall, primary decomposers, secondary decomposers, endophagous species, and predators/scavengers were well represented based on a detailed analysis of species composition at the studied forests (see Section 3: Results and Lu, 2021); stable isotope analysis largely confirmed the a priori ascribed trophic guilds (Supplementary results; Table S1).

We used linear mixed‐effects models (LMMs) to analyze the variation in δ^13^C and δ^15^N values of Oribatida species. Fixed effects included depth (litter and 0–5 cm soil), forest type (European beech, Douglas fir, Norway spruce, mixture of European beech with Douglas fir, mixture of European beech with Norway spruce), guild (primary decomposer, secondary decomposer, endophagous, predatory/scavenging), and site condition (sandy and loamy). All single predictors had variance inflation factors (VIF) below five, suggesting that collinearity is not an issue of concern (O'Brien, 2007). In fixed effects, we included two‐factor interactions of depth x forest type, depth x site condition, depth x guild, and forest type x guild because our focus was to evaluate (1) depth effects and their dependencies on other factors and (2) forest type effects and how they depend on guilds (Table S3). Stable isotope values of litter and of underlying 5 cm depth soil depth were included as covariates at stand level to control for differences in the baseline across forests (Melguizo‐Ruiz et al., 2017). The random effects included 40 forest stands and 40 species, accounting for the non‐independence of samples from the same soil core and to estimate a random intercept for each species. No significant spatial autocorrelation was found based on Moran's I test of model residuals (for both δ^13^C and δ^15^N models, Moran's *I* = −0.166, *p* = .999). The treatment of species as random effects allows to compare response variables at species level as well as at community level.

For bulk material of litter and soil, we modeled their stable isotope values as a function of forest type (European beech, Douglas fir, Norway spruce, European beech/Douglas fir, European beech/Norway spruce), depth (litter and soil), site condition (sandy and loamy), and their interactions. We applied contrasts to inspect differences in δ^13^C and δ^15^N values between litter and soil for Oribatida species as well as for bulk material. The contrast was designated as the difference of estimated marginal means between litter and soil (Piovia‐Scott et al., 2019). We estimated the contrast for each feeding guild and forest type. Furthermore, to quantify differences in δ^13^C and δ^15^N values of Oribatida species between forest types, we compared δ^13^C and δ^15^N values of Oribatida species between forest types using European beech, the climax tree species in Central Europe, as reference (Leuschner et al., 2017; Lu & Scheu, 2021).

All analyses were done in R v4.0.3 (https://www.r‐project.org/). We used “lme4” to fit LMMs (lmer) (Bates et al., 2015) and “emmeans” to estimate marginal means. The package “lmerTest” was used to derive *p*‐values of LMMs with degrees of freedom estimated by Satterthwaite's method (Kuznetsova et al., 2017). All LMMs met the assumptions of normality of residuals and homogeneity of variance.

## 3 RESULTS

**FIGURE 1:**
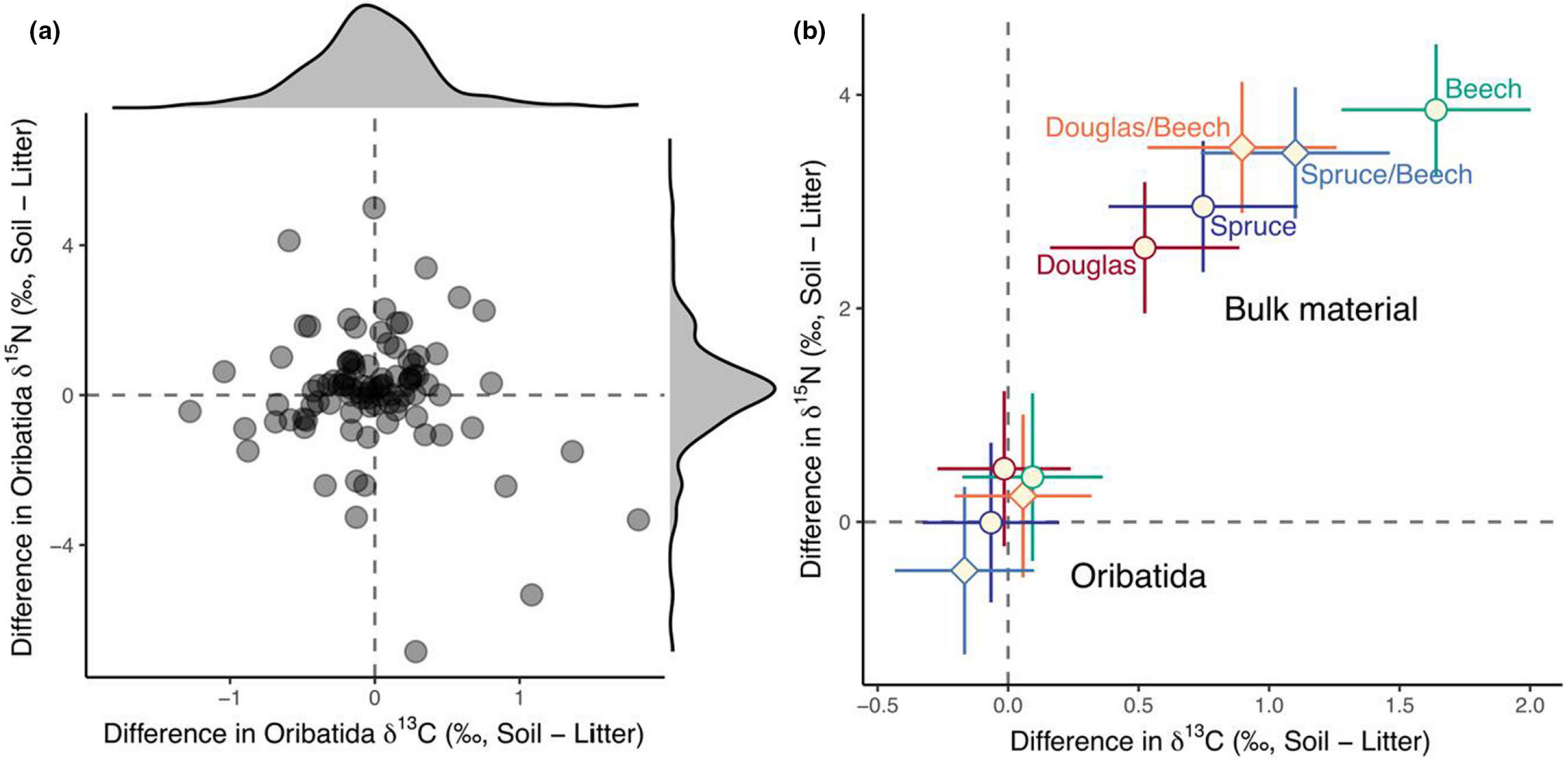
(a) Differences in δ^13^C and δ^15^N values of Oribatida species between soil and litter. Each point represents the isotopic difference between soil and litter for individual species in the same soil core. (b) Differences in δ^13^C and δ^15^N values of mites and bulk material between soil and litter in five forest types [European beech (beech, green), Norway spruce (spruce, blue), Douglas fir (Douglas, red), mixture of Norway spruce and European beech (spruce/beech, light‐blue), mixture of Douglas fir and European beech (Douglas/beech, orange)]; means and 95% confidence intervals.

**FIGURE 2:**
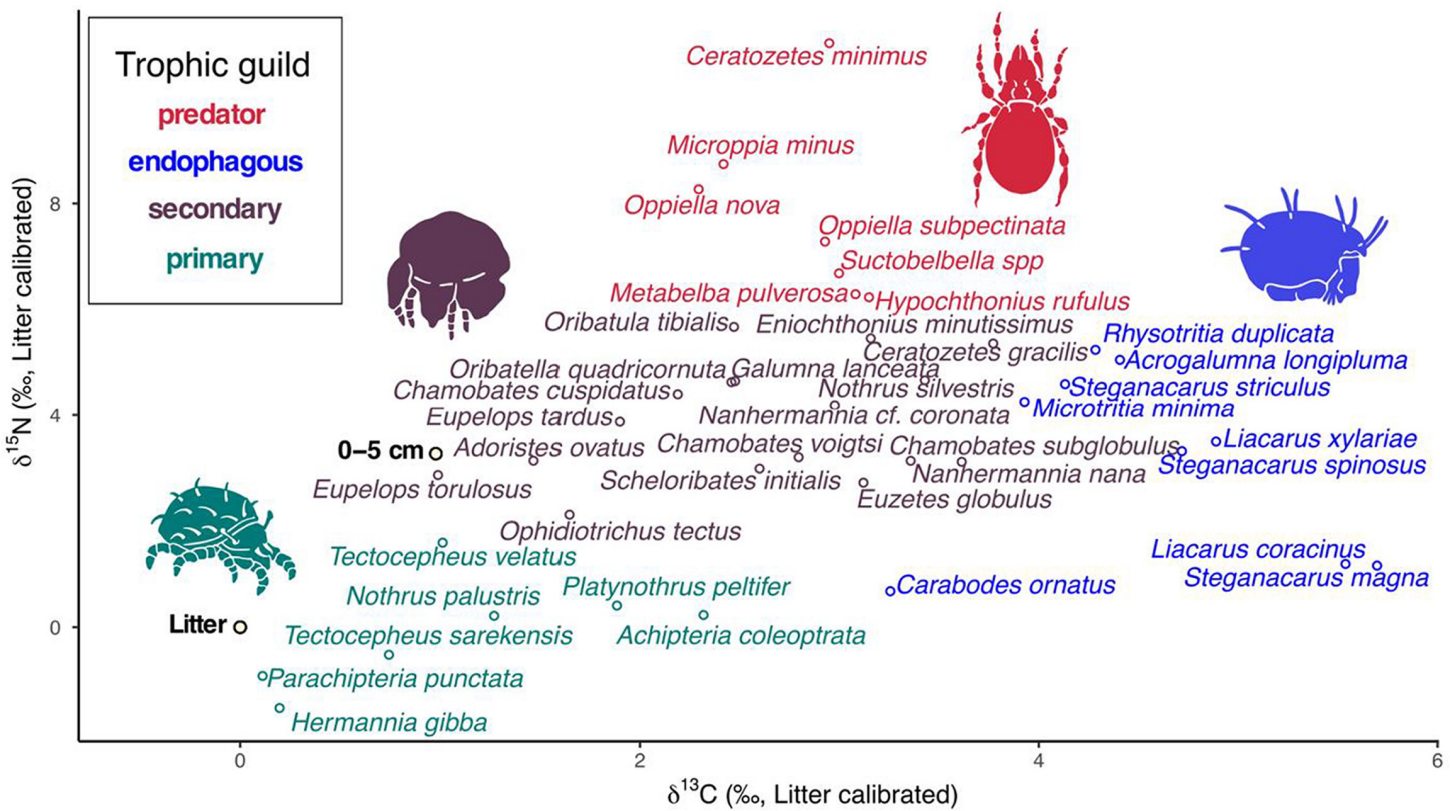
Feeding guilds of Oribatida species assigned based on litter calibrated δ^13^C and δ^15^N values; litter δ^13^C and δ^15^N values were − 28.69 ± 0.67 and −5.59 ± 1.00 (mean ± SD), respectively; feeding guilds are color coded [primary decomposer (green), secondary decomposer (brown), endophagous (blue), and predatory (red)].

**FIGURE 3:**
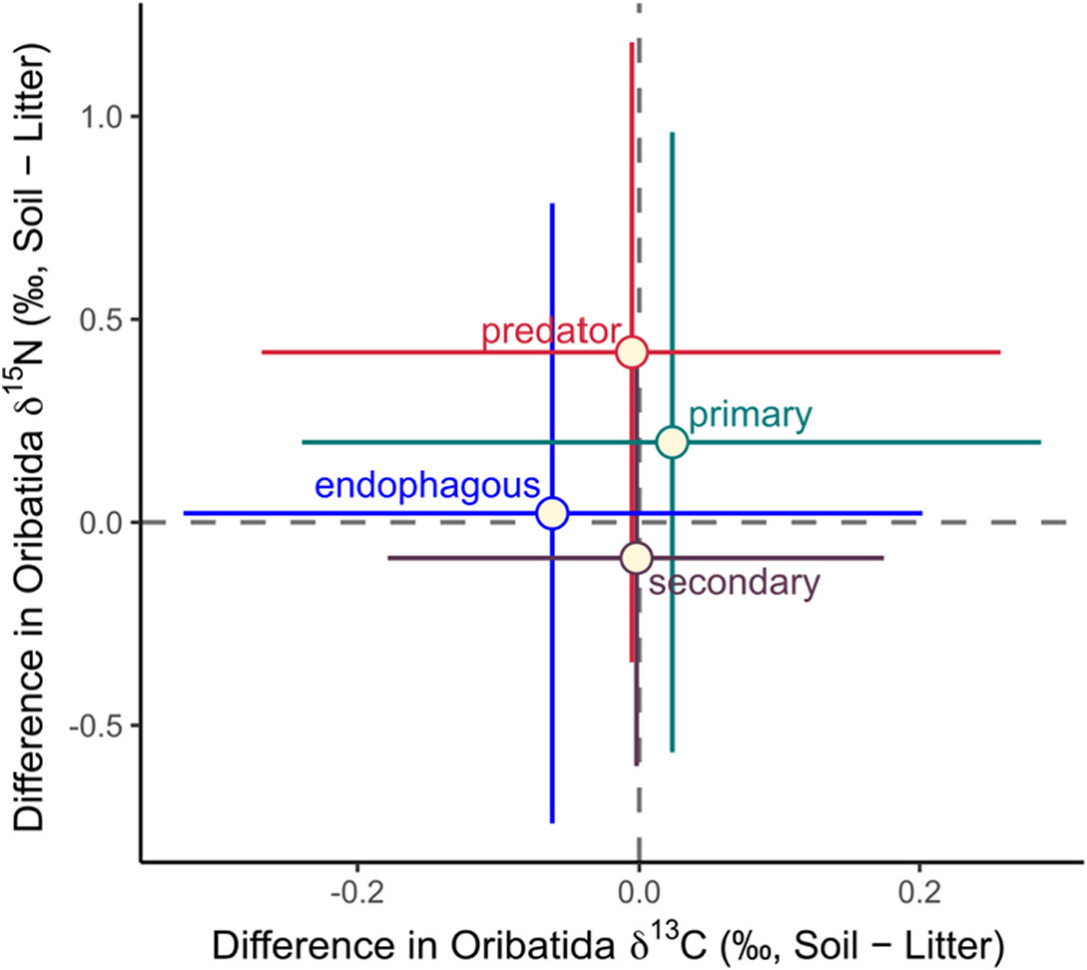
Differences in δ^13^C and δ^15^N values of feeding guilds of Oribatida [primary decomposer (green), secondary decomposer (brown), endophagous (blue), and predatory (red)]; means and 95% confidence intervals.

The difference in δ^13^C and δ^15^N values between litter and soil was highest in European beech (1.64 ± 0.23‰ and 3.85 ± 0.34‰ for δ^13^C and δ^15^N, respectively) and lowest in Douglas fir forests (0.52 ± 0.23‰ and 2.57 ± 0.34‰ for δ^13^C and δ^15^N, respectively). Despite differences in depth gradients of bulk stable isotope values in the studied forests (Table S4, Figure S2), δ^13^C and δ^15^N values of Oribatida species did not differ significantly between litter and soil (Table 1, Figure 1). The selected 40 species covered primary decomposers (*n* = 7), secondary decomposers (*n* = 17), endophagous (*n* = 9), and predatory/scavenging species (*n* = 7; Table S3, Figure 2). The difference in δ^13^C and δ^15^N values of Oribatida species between litter and soil did not depend on the feeding guilds they belong to (Figure 3).

**TABLE 1:** Linear mixed‐effects models on δ^13^C and δ^15^N values of Oribatida species (type III ANOVA)

Factor	δ^13^C Oribatida				δ^15^N Oribatida			
	df	SS	*F*	*p*	df	SS	*F*	*p*
Depth (D)	1,92	0.01	0.02	.880	1,99	0.52	0.39	.534
Forest type (F)	4,35	3.77	**6.86**	**<.001**	4,30	8.34	1.56	.209
Guild (G)	3,30	11.46	**27.82**	**<.001**	3,30	**124.18**	**31.05**	**<.001**
Site condition (S)	1,37	0.94	**6.87**	**.013**	1,31	0.26	0.20	.661
Soil δ^13^C	1,24	0.05	0.39	.537	1,21	0.51	0.38	.542
Litter δ^13^C	1,26	0.14	0.98	.331	1,29	**17.09**	**12.82**	**.001**
D x F	4,92	0.28	0.50	.733	4,99	6.58	1.23	.302
D x G	3,92	0.02	0.04	.990	3,99	0.72	0.18	.910
D x S	1,92	0.29	2.08	.153	1,99	2.61	1.96	.165
F x G	12,106	4.70	**2.85**	**.002**	12,109	19.13	1.20	.295

Fixed effects include depth (litter and soil), Forest type (European beech, Douglas fir, Norway spruce and mixed forests of European beech and Douglas fir and European beech and Norway spruce), guild (primary decomposer, secondary decomposer, endophagous, predatory), site condition (sandy and loamy sites), and interactions. δ values of bulk litter and soil were included as covariates to control for the variation in stable isotope values of basal resources across forest ecosystems. Random effects included 40 forest stands and 40 species. Satterthwaite's method was used to estimate denominator degrees of freedom (df). SS stands for sum of squares.

Significant *p*‐values are in bold (*p* ≤ .05).

**FIGURE 4:**
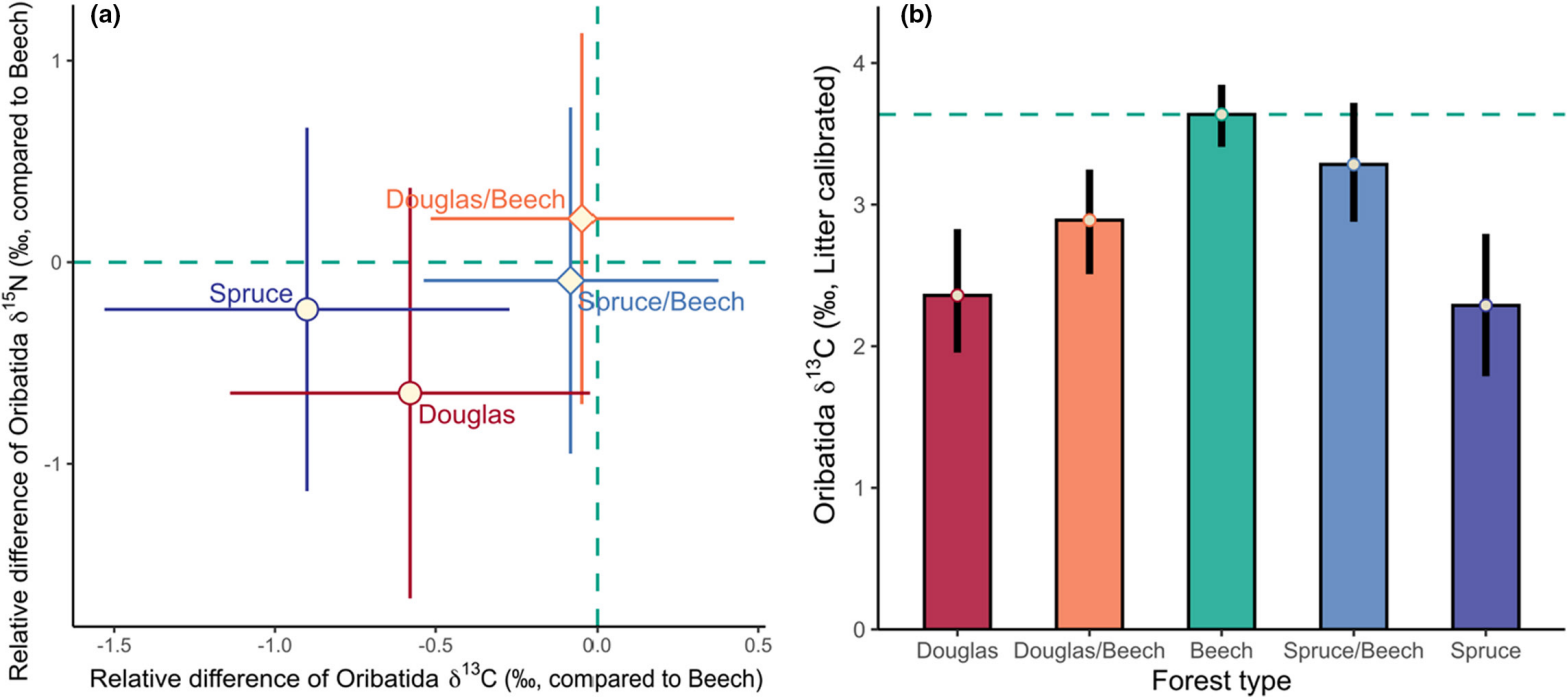
Variations in trophic niches of Oribatida species across forest types. The data include oribatid mites from both litter and soil. (a) Difference in δ^13^C and δ^15^N values of Oribatida species in comparison to European beech forests (beech, green); means and 95% confidence intervals. (b) Comparison of δ^13^C values of Oribatida species between forest types [European beech (beech, green), Douglas fir (Douglas, red), Norway spruce (spruce, blue), Douglas fir and European beech mixture (Douglas/beech, orange), and Norway spruce/European beech mixture (spruce/beech, light‐blue)]; means and 95% confidence interval (estimated by bootstrapping using “mean.cl.Boot”).

**FIGURE 5:**
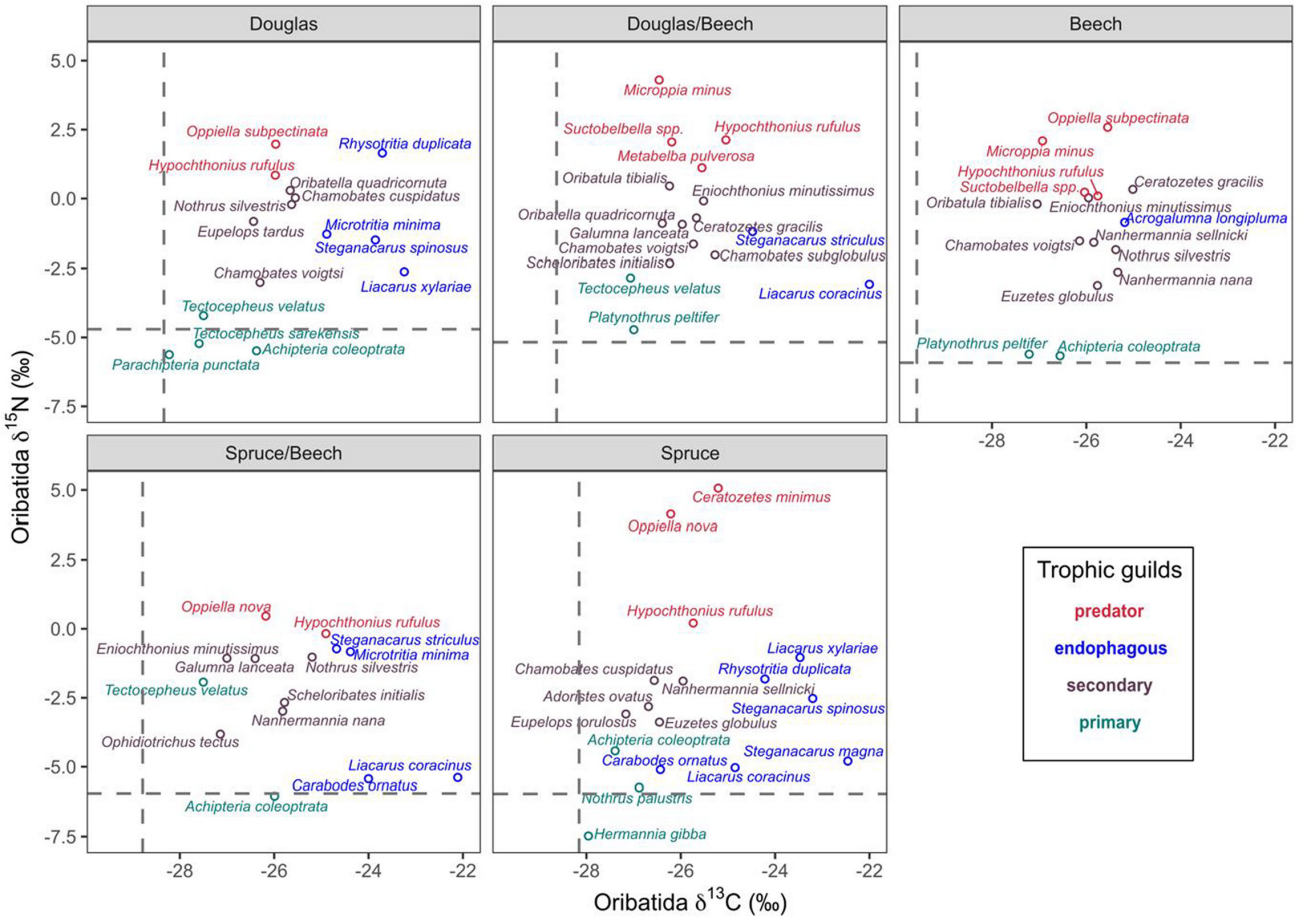
δ^13^C and δ^15^N values of Oribatida species (means) in each of the five forest types studied [Douglas fir (Douglas), mixture of Douglas fir/European beech (Douglas/beech), European beech (beech), mixture of Norway spruce/European beech (spruce/beech), Norway spruce (spruce)]. Dashed lines represent litter δ^13^C and δ^15^N values. Feeding guilds are color coded: Primary decomposer (green), secondary decomposer (brown), endophagous (blue), and predatory species (red).

To account for variations in δ^13^C and δ^15^N values in basal resources across forest types, we included bulk stable isotope values of litter and soil as covariates; δ^15^N values of Oribatida species varied stronger with δ^15^N values of litter than with those of soil across forests (Table 1; *F*_1,29_ = 12.82, *p* = .001 for the effect of Litter). Further, δ^13^C values of Oribatida species were higher in European beech than in Norway spruce (0.90‰) and Douglas fir forests (0.58‰ Figure 4, Figure S3). The δ13C enrichment of Oribatida guilds in European beech forests was significant in secondary decomposer Oribatida, but not in primary decomposer and predatory/scavenging Oribatida (interaction of Forest type and Trophic guild; Table 1); δ^13^C enrichment in endophagous Oribatida was higher in mixed forests than in European beech forests (Figure S4). By contrast, as reflected by δ^15^N values, the trophic position of Oribatida species did not differ significantly between forest types (Table 1, Figures 4a and 5, Figure S3).

## 4 DISCUSSION

To better understand trophic consistency in species, we compared trophic niches of Oribatida mites by using stable isotope analyses in litter and soil across forest types. We found that mean trophic niches of Oribatida species, as indicated by their δ^13^C and δ^15^N values, are highly consistent between litter and soil, and their trophic positions (δ^15^N values) are similar across deciduous and coniferous forests. Our results suggest that trophic niches of Oribatida species vary little with the habitat they live in and the associated environmental conditions. This low intraspecific variation combined with pronounced interspecific differences in trophic niches may facilitate coexistence of soil Oribatida species (Hart et al., 2016; Schneider et al., 2004).

### 4.1 Variations between litter and soil

In contrast to strong depth‐isotope gradients in bulk materials of the studied forests, Oribatida species occupied remarkably consistent trophic niches irrespective of soil depth. This rejects our first hypothesis and stresses that the actual depth which Oribatida species are colonizing does not inform us about where the majority of food resources are acquired during their lifetime (Scheu & Falca, 2000). As indicated by the depth‐isotope gradients of bulk litter and soil, the decomposition rate and speed of nitrogen cycling differed between European beech and coniferous forests. We sampled soil down to 5 cm depth, a gradient equivalent to ca. 50–200 times the body length of Oribatida. The consistent trophic niches between litter and soil may point towards high vertical mobility of Oribatida species (Åström & Bengtsson, 2011; Brückner et al., 2018), but also to the presence of their trophic niches irrespective of soil depth in the forest floor. In any case, the high consistency of trophic niches in Oribatida species underlines the importance of niche differentiation for the coexistence of Oribatida species in soil (Schneider et al., 2004).

Soil Oribatida have diversified into multitrophic levels in soil food webs, forming a spectrum from primary decomposer to predators/scavengers (Scheu & Falca, 2000; Schneider et al., 2004). We investigated four Oribatida guilds representing their trophic diversity in soil food webs (Maraun et al., 2011; Schneider et al., 2004). The availability of food resources changes rapidly with soil depth, with higher proportions of bacteria and mycorrhizal fungi deeper in soil relative to saprotrophic fungi, which dominate at earlier stages of decay in litter (Lindahl et al., 2007; Lu & Scheu, 2021). In contrast to our second hypothesis, the high consistency of trophic niches across litter and soil in Oribatida species applied to each of the four guilds (Table 1, Figures 1 and 3). Our findings also challenge the view that Oribatida species are opportunistic feeders (Maraun & Scheu, 2000); rather the results suggest that they occupy distinct niches in the field irrespective of the soil depth they inhabit. Notably, this applies to a wide range of species including major feeding guilds of Oribatida, colonizing pure and mixed forests of deciduous and coniferous trees, arguing for the generality of these results.

### 4.2 Variations with forest type

The detrital shift, that is, the enrichment in δ^13^C values relative to litter (Pollierer et al., 2009; Susanti et al., 2021), has been widely documented in terrestrial ecosystems, suggesting that it is a universal phenomenon in decomposer food webs (Potapov et al., 2019). We found that the detrital shift is stronger in European beech than in coniferous forests. This differential shift suggests that the basal resources of Oribatida species differ between forest types, supporting our third hypothesis. Two non‐mutually exclusive mechanisms may explain the differences in the detrital shift. First, litter of European beech may be low in quality and rich in (^13^C depleted) lignin resulting in saprotrophic microorganisms to preferentially incorporate palatable litter compounds that are enriched in ^13^C (Pollierer et al., 2009). Indeed, δ^13^C values of leaf litter of European beech (−29.58 ± 0.33‰) were about 1.27–1.41‰ lower than those of needle litter of coniferous trees (−28.31 ± 0.37‰ and − 28.17 ± 0.44‰ for Douglas fir and Norway spruce, respectively; means ± SD). Further, high microbial activity in European beech forests accelerates carbon turnover and increases the incorporation of microbial processed carbon into soil food webs (Potapov et al., 2019), presumably contributing to the more pronounced detrital shift in European beech compared to coniferous forests. Indeed, as indicated by microbial biomass and microbial basal respiration, microbial activity in European beech forests exceeds that in coniferous forests, which may be due to more efficient decomposer communities and/or favorable abiotic conditions (Albers et al., 2004; Lu & Scheu, 2021). Supporting the importance of microbial activity, differences in the detrital shift between European beech and coniferous forests were strongest in secondary decomposer Oribatida, known to predominantly feed on fungi (Pollierer & Scheu, 2021; Schneider et al., 2004). Our findings on the detrital shift likely also apply to other soil invertebrates feeding on microorganisms reflecting differential resource use in the respective soil food webs.

Although basal resources differ between European beech and coniferous forests, our results suggest that the trophic position of Oribatida species is consistent irrespective of forest types, contrasting our third hypothesis. Recent studies indicated that Douglas fir detrimentally affects soil microbial communities at nutrient‐poor sites as well as the community composition of Oribatida (Lu, 2021). However, as shown in the present study, trophic positions of Oribatida species are little affected by Douglas fir. This suggests that tree species either little affect the trophic structure of Oribatida and potentially also other detritivores (Díaz‐Aguilar & Quideau, 2013; Pollierer et al., 2021) or that non‐native tree species provide very similar or even the same trophic niches for Oribatida species as native tree species. In tropical ecosystems ranging from rainforest to monoculture plantations, some Oribatida species shift their trophic niches as reflected by δ^15^N (Krause et al., 2019), which we could not confirm for Oribatida species in temperate forests. Rather, our results agree with recent studies based on variations in stable isotope ratios highlighting the consistency of trophic niches of other mesofauna groups, such as Mesostigmata mites and Collembola, between different land‐use systems (Klarner et al., 2017; Susanti et al., 2021). The paradox of opportunistic feeding at laboratory conditions but invariant trophic positions in the field calls for more studies on the microhabitats and foraging ecology of soil microarthropod species (Brückner et al., 2018; Erktan et al., 2020; Tordoff et al., 2008).

### 4.3 Oribatida species and guilds

High trophic level Oribatida species (i.e., predators/scavengers) on average were enriched in δ^15^N by 6.7–11.0‰, consistent with the enrichment of other predators in temperate forests including Mesostigmata mites, Chilopoda, and Araneida (Pollierer et al., 2009; Potapov et al., 2019). Incorporation of old organic matter in detritivores may inflate their high trophic positions. Arguing against this mechanism, δ^15^N values of Oribatida species correlated more closely with δ^15^N values of litter than with those of soil, suggesting that Oribatida species predominantly rely on fresh organic matter resources in litter or on root‐derived resources irrespective of the actual soil depth they inhabit (Melguizo‐Ruiz et al., 2017; Okuzaki et al., 2009; Pollierer et al., 2007). Therefore, tissue carbon and nitrogen of predatory/scavenging Oribatida is unlikely to be derived from old organic matter, contrasting other mesofauna with high δ^15^N values such as euedaphic Collembola (Li et al., 2021; Potapov et al., 2019). In addition to living as predator/scavenger, species such as *Oppiella nova*, the most abundant soil mesofauna species at our study sites, may also feed on ectomycorrhizal fungi, known to be enriched in ^15^ N (Potapov & Tiunov, 2016; Remén et al., 2008). The actual resource used by *O. nova* and other species of the predator/scavenger guild of Oribatida needs further attention including experiments manipulating the input of root‐derived resources (Bluhm et al., 2021).

We assigned Oribatida species to feeding guilds based on their average values of δ^13^C and δ^15^N. This approach assumes that the grouping is independent of forest types which was confirmed by our results. The high consistency of trophic positions of Oribatida species has important implications. It allows ascribing Oribatida species to feeding guilds based on their δ^15^N values, which helps to reduce the complexity of species‐based soil food webs while keeping the multitrophic structure of Oribatida (Nielsen, 2019). Future research disentangling the channeling of different basal resources into these feeding guilds may help to uncover the food resources they are actually feeding on (Pollierer & Scheu, 2021; Scheu, 2002). Further, the guild approach facilitates the comparison of Oribatida communities across forest ecosystems as their trophic position may be used as a quantitative trait in trait‐based community analyses (Moosmann et al., 2021; Simberloff & Dayan, 1991; Violle et al., 2007). Overall, our sampling design and statistical approach provided detailed insight into the trophic structure of a wide range of Oribatida species and feeding guilds across ecosystems. The results also call for studying the vertical variation of trophic niches in other soil animal groups across different environments to move towards more context‐explicit trophic interactions in soil food webs.

## AUTHOR CONTRIBUTIONS

**Jing‐Zhong Lu:** Conceptualization (equal); data curation (lead); formal analysis (lead); investigation (lead); methodology (equal); project administration (lead); resources (equal); software (lead); supervision (lead); validation (lead); visualization (lead); writing – original draft (lead); writing – review and editing (equal). **Peter Hans Cordes:** Investigation (equal); writing – review and editing (supporting). **Mark Maraun:** Conceptualization (supporting); investigation (supporting); methodology (supporting); resources (supporting); supervision (supporting); validation (equal); visualization (supporting); writing – original draft (supporting); writing – review and editing (equal). **Stefan Scheu:** Conceptualization (equal); funding acquisition (lead); methodology (supporting); project administration (supporting); resources (lead); supervision (lead); validation (supporting); writing – review and editing (lead).

## FUNDING INFORMATION

This work was supported by the German Research Foundation (Grant ID: 316045089 & 458736525).

## Supporting information


Appendix S1

## Data Availability

The data that supports the findings of this study are available in the supplementary material of this article.
