# Supplementary material for: High consistency of trophic niches in generalist arthropod species (Oribatida, Acari) across soil depth and forest type
Source: Ecol Evol. 2022 Dec 12;12(12):e9572. doi: 10.1002/ece3.9572 (PMC9745105; doi:10.1002/ece3.9572)
Supplement: Supplementary file 1 — Appendix S1 [file ECE3-12-e9572-s001.docx]

**Supplementary materials**

High consistency of trophic niches in generalist arthropod species (Oribatida, Acari) across soil depth and forest type

**Authors**: Jing-Zhong Lu^1*^, Peter Hans Cordes^1^, Mark Maraun^1^, Stefan Scheu^1,2^

**Affiliations**:

1. Johann-Friedrich-Blumenbach Institute of Zoology and Anthropology, Universität Göttingen, Untere Karspüle 2, 37073 Göttingen, Germany

2. Center of Biodiversity and Sustainable Land Use, Universität Göttingen, Büsgenweg 1, 37077 Göttingen, Germany

* Corresponding author (E-mail: [jlu@gwdg.de](mailto:jlu@gwdg.de))

# Table of Contents

**Supplementary Results**

On guild ascription

**Supplementary Figures**

Fig. S1. Diagram of study design

Fig. S2. Depth gradients of bulk stable isotopes

Fig. S3. Comparison in isotopic niches across forest types

Fig. S4. Guild-specific forest-type effects on trophic niches of oribatid mites

**Supplementary Tables**

Table S1. Sampling design and feeding guild ascription

Table S2. Mean and standard error of bulk stable isotope values of 40 oribatid mite species

Table S3. Sampling design for number of samples in each feeding guilds

Table S4. F and P values of linear mixed effect models on bulk stable isotope values

**Supplementary References**

# Supplementary Results

## On guild ascription

We evaluated guild ascription based on measured data, and this resulted in a change of eight species. However, among the eight species, four were due to a lack of published species-level data (*Ceratozetes minimus*, *Metabelba pulverosa*, *Carabodes ornatus*, *Adoristes ovatus*), so our inference was based on genus or family data of stable isotope values (Table S1). This is not surprising, because trophic position has little phylogenetic signal, so the trophic position of related species does not tend to be similar (Schaefer & Caruso 2019). Another species (*Acrogalumna longipluma*) was ascribed from secondary to endophagous (a group that incorporate CaCO_3_), and this is only due to changes of ^13^C but not likely of ^15^N, agreeing with our findings that ^13^C is affected by forest types, but ^15^N is highly consistent (Fig. 4). The remaining three species were changed from primary decomposers to secondary decomposers (*Euzetes globulus*, *Ophidiotrichus tectus*, *Oribatella quadricornuta*). Given that guilds are continuous categories, we do not think such change is significant and influences our statistic results and conclusions. Further, the purpose of guild approach in this study is not to show how guilds capture variation in isotope values, but to serve as a trait of species when testing interactions with environmental gradients of interests, especially soil depth and forest type. We ascribed guilds *a priori* so that we can select species that well represent their trophic diversity. This is in fact a strength of this study, as many small predator/scavengers oribatid mites were sampled. Therefore, we used re-ascribed guilds based on measured data, which is always recommended (Potapov et al., 2019).

# Supplementary Figures

## Figure S1


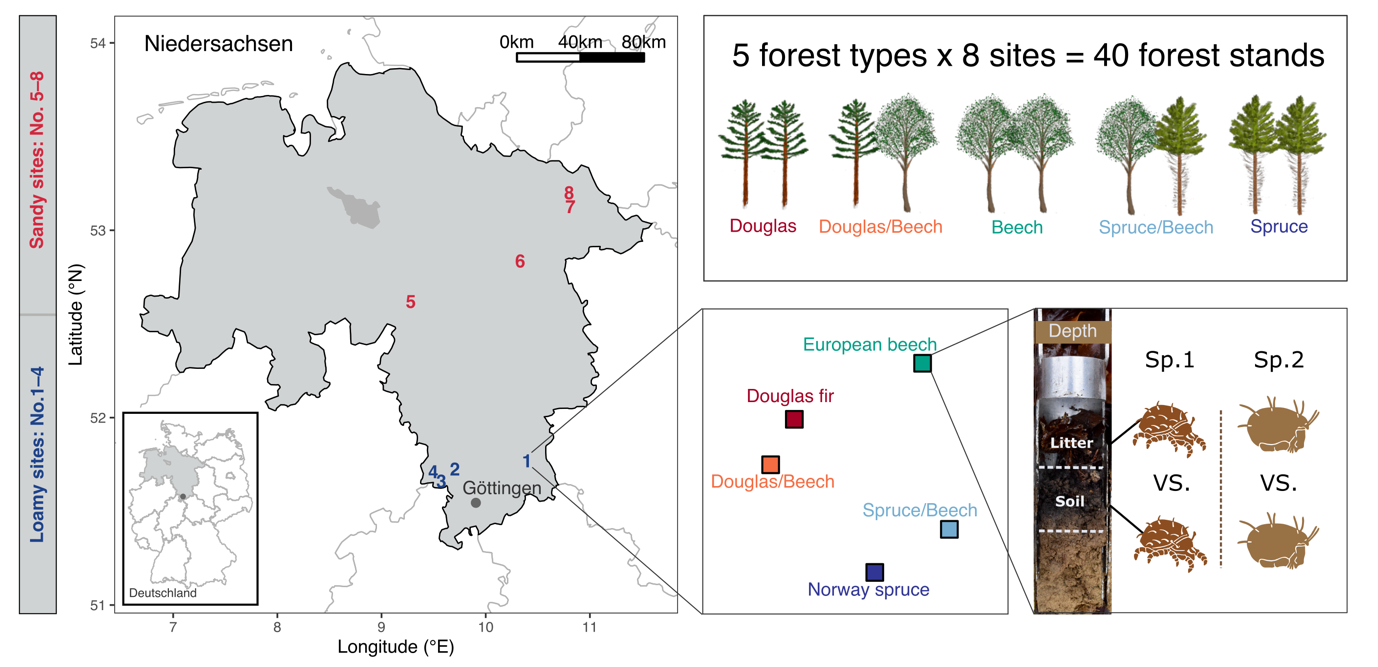


Figure S1. Study design and locations of study sites. The study sites include four southern sites on loamy soils (1–4) and four northern sites on sandy soils (5–8). At each site, five forest types were investigated including European beech (Beech), Norway spruce (Spruce), Douglas fir (Douglas), mixture of Norway spruce and European beech, and mixture of Douglas fir and European beech. Soil cores were separated into litter (O_L_) and 0–5 cm depth (Soil, mainly O_F/H_, A_H_). Two to three Oribatida species were selected which occurred in both litter and soil.

## Figure S2


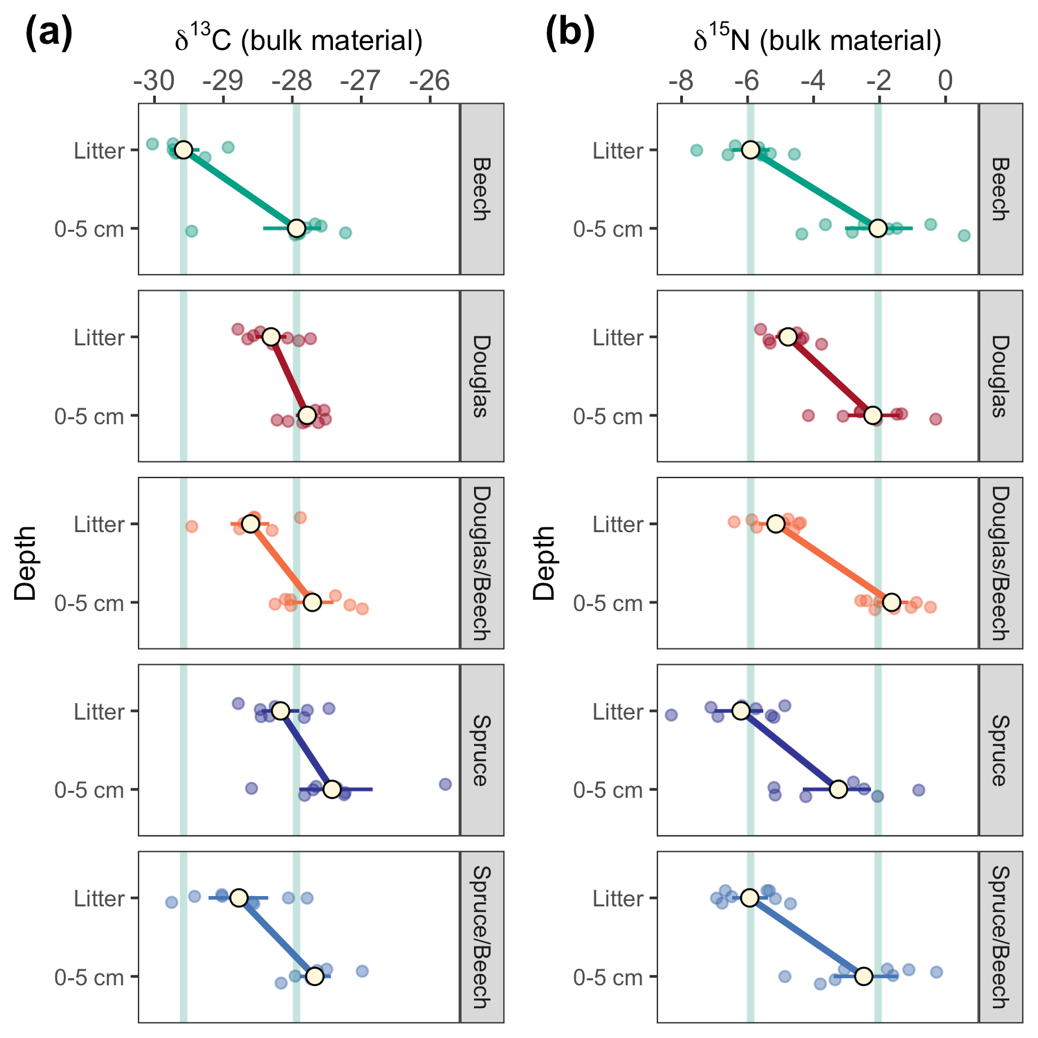


**Figure S2**. Stable isotope values of δ^13^C (a) and δ^15^N (b) of bulk litter and 0–5 cm soil in European beech (Beech), Douglas fir (Douglas), Douglas fir/European beech (Douglas/Beech), Norway spruce (Spruce), Norway spruce/European beech (Spruce/Beech) forests. Horizontal bars are bootstrap estimated standard errors (n = 8). The green vertical bars represent respective values in beech forests in litter and 0–5 cm soil.

Figure S4


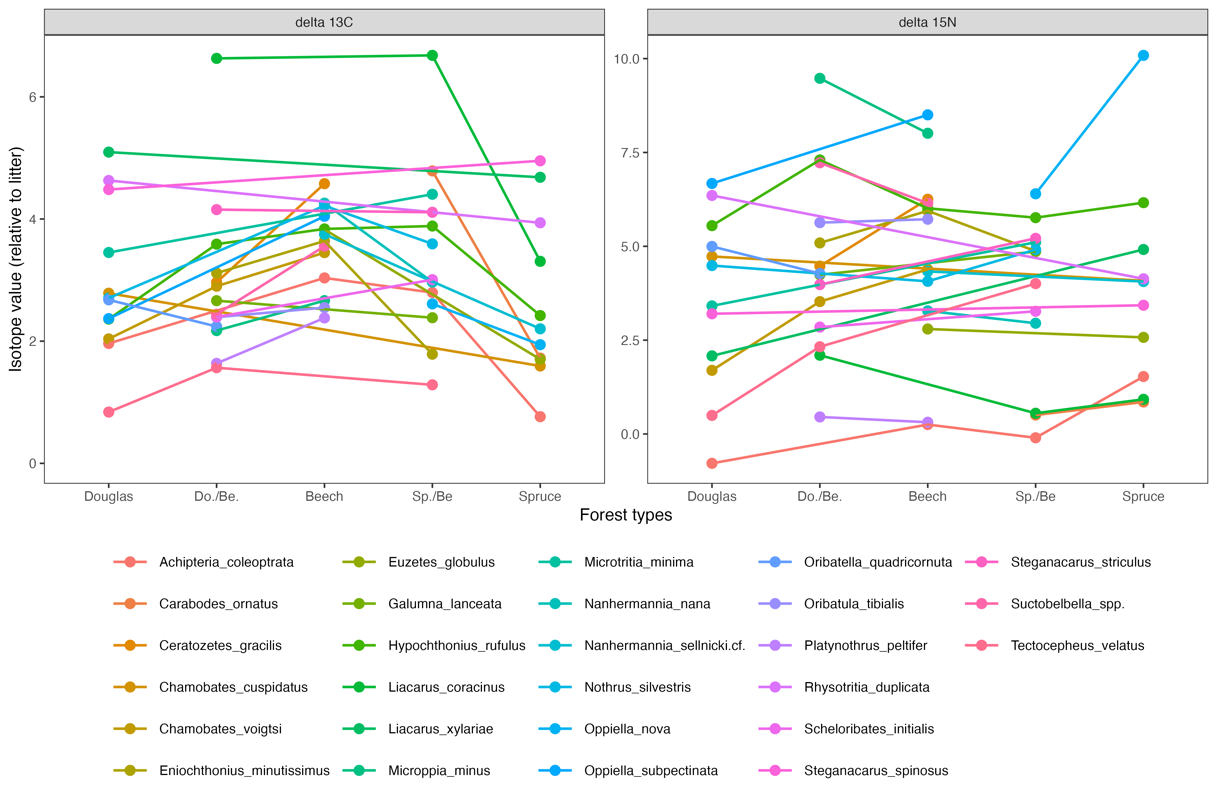


**Figure S3**. Differences in litter calibrated mean δ^13^C and δ^15^N values between forest types. Analysis of a subset of data including 27 oribatid mite species sampled in two or more forest types. The five forest types are pure Douglas fir (Douglas), European beech (Beech), Norway spruce (Spruce) and mixtures of Douglas fir and European beech (Do./Be.), and Norway spruce and European beech (Sp./Be.).

## Figure S4


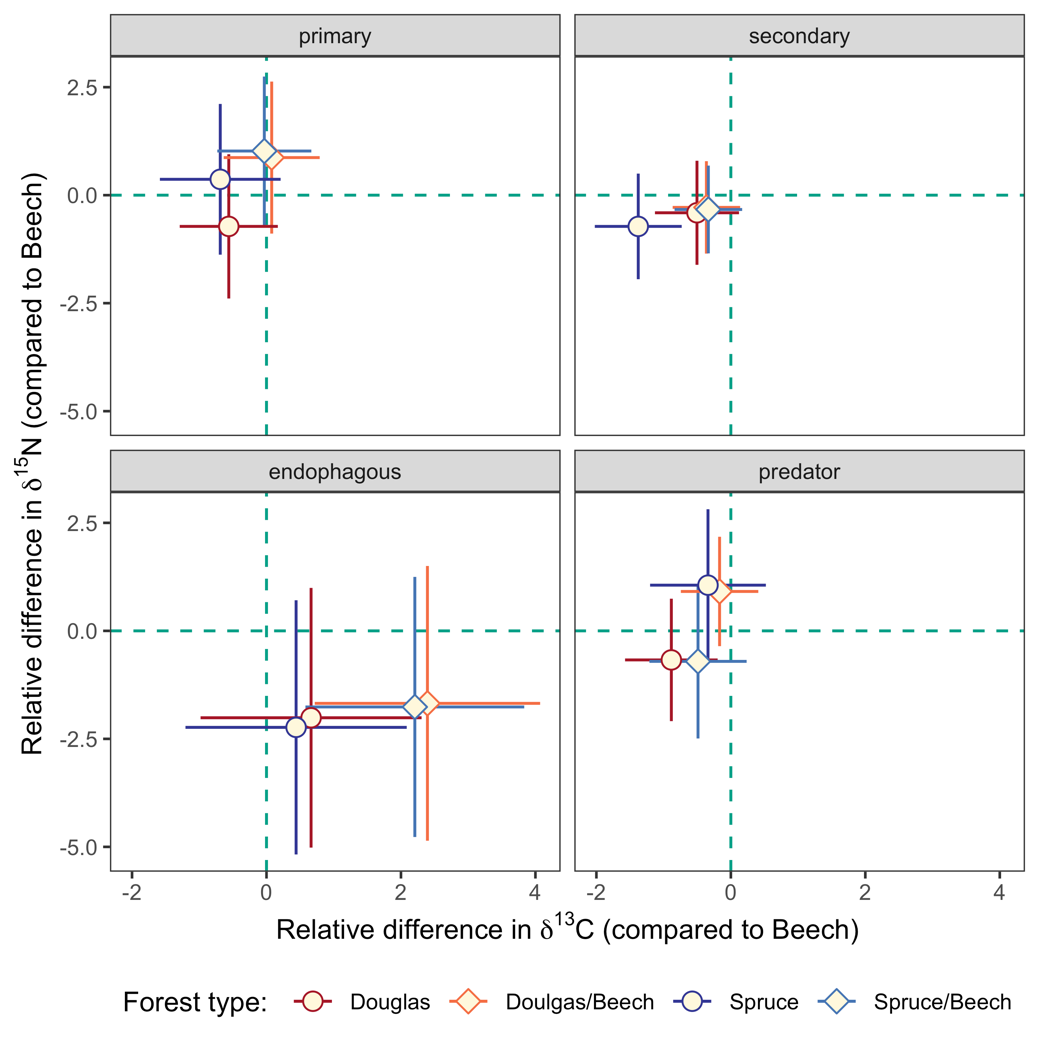


**Figure S4**. Difference in δ^13^C and δ^15^N values of Oribatida guilds (primary decomposer, secondary decomposer, endophagous and predatory) in comparison to European beech forests (Beech, dash line in green); Forest types include Douglas fir (Douglas, red), Norway spruce (Spruce, blue), Douglas fir and European beech mixture (Douglas/Beech, orange) and Norway spruce/European beech mixture (Spruce/Beech, light-blue); means and 95 % confidence intervals.

# Supplementary Tables

## Table S1

Sampling design and feeding guilds of Oribatida. Species were ascribed to guilds [primary decomposers, secondary decomposers, endophagous, predators/scavengers] based on literature (genus and family were noted in the reference when no matched species), and then re-ascribed based on stable isotope measurements of the present study (ascribed guild underlined). For each species, number of populations in each forest type is given and how many forest types the species was investigated from is summarized (occurrence in forest types). The forest types studied included Douglas fir (Do), Douglas/Beech mixture (Do/Be), European beech (Be), Spruce/Beech mixture (Sp/Be), Norway spruce (Sp). Species selected in more than one forest type were marked in green under forest types.

| Guild | Oribatida taxa | Ascribed guild | Reference | Do | Do/Be | Be | | Sp/Be | | Sp | | Occurrence in forest type | |  |
| --- | --- | --- | --- | --- | --- | --- | --- | --- | --- | --- | --- | --- | --- | --- |
| predator | *Ceratozetes minimus* | secondary | Maraun et al. 2011, genus | 0 | 0 | 0 | | 0 | | 2 | | 1 | | |
| predator | *Hypochthonius rufulus* | predator | Maraun et al. 2011 | 4 | 4 | 2 | | 2 | | 2 | | 5 | | |
| predator | *Metabelba pulverosa* | secondary | Lehmitz et al. 2016, genus | 0 | 2 | 0 | | 0 | | 0 | | 1 | | |
| predator | *Microppia minus* | predator | Maraun et al. 2011, family | 0 | 2 | 2 | | 0 | | 0 | | 2 | | |
| predator | *Oppiella nova* | predator | Maraun et al. 2011, family | 0 | 0 | 0 | | 2 | | 2 | | 2 | | |
| predator | *Oppiella subpectinata* | predator | Maraun et al. 2011, family | 4 | 0 | 2 | | 0 | | 0 | | 2 | | |
| predator | *Suctobelbella* spp. | predator | Schneider et al. 2004 | 0 | 2 | 2 | | 0 | | 0 | | 2 | | |
| endophagous | *Acrogalumna longipluma* | secondary | Magilton et al. 2019 | 0 | 0 | 4 | | 0 | | 0 | | 1 | | |
| endophagous | *Carabodes ornatus* | secondary | Bluhm et al. 2015, genus | 0 | 0 | 0 | | 2 | | 2 | | 2 | | |
| endophagous | *Liacarus coracinus* | endophagous | Bluhm et al. 2015 | 0 | 2 | 0 | | 2 | | 2 | | 3 | | |
| endophagous | *Liacarus xylariae* | endophagous | Schneider et al. 2004, genus | 2 | 0 | 0 | | 0 | | 2 | | 2 | | |
| endophagous | *Microtritia minima* | endophagous | Magilton et al. 2019, family | 2 | 0 | 0 | | 2 | | 0 | | 2 | | |
| endophagous | *Rhysotritia duplicata* | endophagous | Schneider et al. 2004 | 2 | 0 | 0 | | 0 | | 2 | | 2 | | |
| endophagous | *Steganacarus magna* | endophagous | Maraun et al. 2011 | 0 | 0 | 0 | | 0 | | 2 | | 1 | | |
| endophagous | *Steganacarus spinosus* | endophagous | Schneider et al. 2004 | 2 | 0 | 0 | | 0 | | 2 | | 2 | | |
| endophagous | *Steganacarus striculus* | endophagous | Schneider et al. 2004 | 0 | 2 | 0 | | 2 | | 0 | | 2 | | |
| secondary | *Adoristes ovatus* | endophagous | Maraun et al. 2011, family | 0 | 0 | 0 | | 0 | | 4 | | 1 | | |
| secondary | *Ceratozetes gracilis* | secondary | Maraun et al. 2011 | 0 | 2 | 2 | | 0 | | 0 | | 2 | | |
| secondary | *Chamobates cuspidatus* | secondary | Maraun et al. 2011 | 2 | 0 | 0 | | 0 | | 2 | | 2 | | |
| secondary | *Chamobates subglobulus* | secondary | Maraun et al. 2011, genus | 0 | 2 | 0 | | 0 | | 0 | | 1 | | |
| secondary | *Chamobates voigtsi* | secondary | Maraun et al. 2011 | 2 | 2 | 2 | | 0 | | 0 | | 3 | | |
| secondary | *Eniochthonius minutissimus* | secondary | Schneider et al. 2004 | 0 | 2 | 2 | | 2 | | 0 | | 3 | | |
| secondary | *Eupelops tardus* | secondary | Maass et al. 2015 | 2 | 0 | 0 | | 0 | | 0 | | 1 | | |
| secondary | *Eupelops torulosus* | secondary | Maraun et al. 2011, genus | 0 | 0 | 0 | | 0 | | 2 | | 1 | | |
| secondary | *Euzetes globulus* | primary | Schneider et al. 2004 | 0 | 0 | 4 | | 0 | | 2 | | 2 | | |
| secondary | *Galumna lanceata* | secondary | Maraun et al. 2011 | 0 | 2 | 0 | | 4 | | 0 | | 2 | | |
| secondary | *Nanhermannia nana* | secondary | Schneider et al. 2004 | 0 | 0 | 2 | | 2 | | 0 | | 2 | | |
| secondary | *Nanhermannia* cf. *coronata* | secondary | Schneider et al. 2004 | 0 | 0 | 2 | | 0 | | 2 | | 2 | | |
| secondary | *Nothrus silvestris* | secondary | Maraun et al. 2011 | 4 | 0 | 2 | | 8 | | 0 | | 3 | | |
| secondary | *Ophidiotrichus tectus* | primary | Magilton et al. 2019 | 0 | 0 | 0 | | 2 | | 0 | | 1 | | |
| secondary | *Oribatella quadricornuta* | primary | Schneider et al. 2004 | 2 | 2 | 0 | | 0 | | 0 | | 2 | | |
| secondary | *Oribatula tibialis* | secondary | Schneider et al. 2004 | 0 | 2 | 2 | | 0 | | 0 | | 2 | | |
| secondary | *Scheloribates initialis* | secondary | Schneider et al. 2004 | 0 | 4 | 0 | | 2 | | 0 | | 2 | | |
| primary | *Achipteria coleoptrata* | primary | Maraun et al. 2011 | 2 | 0 | 4 | | 2 | | 2 | | 4 | | |
| primary | *Hermannia gibba* | primary | Pollierer et al. 2009 | 0 | 0 | 0 | | 0 | | 4 | | 1 | | |
| primary | *Nothrus palustris* | primary | Maraun et al. 2011 | 0 | 0 | 0 | | 0 | | 2 | | 1 | | |
| primary | *Parachipteria punctata* | primary | Bluhm et al. 2015 | 2 | 0 | 0 | | 0 | | 0 | | 1 | | |
| primary | *Platynothrus peltifer* | primary | Maraun et al. 2011 | 0 | 4 | 2 | | 0 | | 0 | | 2 | | |
| primary | *Tectocepheus sarekensis* | primary | Maraun et al. 2011 | 4 | 0 | 0 | | 0 | | 0 | | 1 | | |
| primary | *Tectocepheus velatus* | primary | Maraun et al. 2011 | 4 | 2 | 0 | | 2 | | 0 | | 3 | | |
| **Total number of species:** | |  |  | 12 | 14 | | 14 | | 13 | | 11 | | 27 | |

## Table S2

Species list of Oribatida (n = 40). Feeding guilds were assigned according to litter calibrated δ^13^C and δ^15^N values: primary decomposer, secondary decomposer, endophagous Oribatida and scavenger/predator. Total number of animals for each species used for stable isotopes and their ranges (min - max) are given.

| Oribatid taxa | Family | Total number (range) | δ^13^C | δ^15^N | Feeding guild |
| --- | --- | --- | --- | --- | --- |
| *Ceratozetes minimus* Sellnick, 1928 | Ceratozetidae | 10 (10-10) | 2.95 ± 0.06 | 11.02 ± 0.17 | predator |
| *Hypochthonius rufulus* C.L. Koch, 1835 | Hypochthoniidae | 4 (2-7) | 3.15 ± 0.77 | 6.23 ± 0.96 | predator |
| *Metabelba pulverosa* Strenzke, 1953 | Damaeidae | 3 (3-3) | 3.08 ± 0.25 | 6.29 ± 2.40 | predator |
| *Microppia minus* (Paoli, 1908) | Oppiidae | 19 (7-25) | 2.42 ± 0.28 | 8.74 ± 2.42 | predator |
| *Oppiella nova* (Oudemans, 1902) | Oppiidae | 12 (8-16) | 2.28 ± 0.44 | 8.24 ± 2.27 | predator |
| *Oppiella subpectinata* (Oudemans, 1900) | Oppiidae | 9 (3-16) | 2.93 ± 0.93 | 7.28 ± 1.96 | predator |
| *Suctobelbella* spp Jacot, 1937 | Suctobelbidae | 22 (18-26) | 3.00 ± 0.74 | 6.69 ± 0.72 | predator |
| *Acrogalumna longipluma* (Berlese, 1904) | Galumnidae | 4 (3-5) | 4.41 ± 0.18 | 5.06 ± 0.12 | endophagous |
| *Carabodes ornatus* Storkan, 1925 | Carabodidae | 2 (1-3) | 3.26 ± 1.79 | 0.68 ± 0.52 | endophagous |
| *Liacarus coracinus* (C.L. Koch, 1841) | Liacaridae | 1 (1-2) | 5.54 ± 1.92 | 1.19 ± 1.37 | endophagous |
| *Liacarus xylariae* (Schrandk, 1803) | Liacaridae | 2 (1-2) | 4.89 ± 0.26 | 3.50 ± 1.98 | endophagous |
| *Microtritia minima* (Berlese, 1904) | Euphthiracaridae | 12 (10-15) | 3.93 ± 0.56 | 4.26 ± 1.05 | endophagous |
| *Rhysotritia duplicata* (Grandjean, 1953) | Euphthiracaridae | 8 (8-9) | 4.28 ± 0.46 | 5.24 ± 1.49 | endophagous |
| *Steganacarus magnus* (Nicolet, 1855) | Phthiracaridae | 1 (1-1) | 5.70 ± 0.00 | 1.16 ± 0.18 | endophagous |
| *Steganacarus spinosus* (Sellnick, 1920) | Phthiracaridae | 4 (1-8) | 4.72 ± 0.36 | 3.32 ± 0.38 | endophagous |
| *Steganacarus striculus* (C.L. Koch, 1835) | Phthiracaridae | 4 (3-5) | 4.13 ± 0.26 | 4.60 ± 0.82 | endophagous |
| *Adoristes ovatus* (C.L. Koch, 1839) | Liacaridae | 1 (1-1) | 1.47 ± 0.58 | 3.14 ± 1.63 | secondary |
| *Ceratozetes gracilis* (Michael, 1884) | Ceratozetidae | 8 (6-10) | 3.77 ± 0.93 | 5.36 ± 1.03 | secondary |
| *Chamobates cuspidatus* (Michael, 1884) | Chamobatidae | 4 (2-7) | 2.19 ± 0.70 | 4.41 ± 0.43 | secondary |
| *Chamobates subglobulus* (Oudemans, 1900) | Chamobatidae | 1 (1-1) | 3.36 ± 0.57 | 3.13 ± 0.22 | secondary |
| *Chamobates voigtsi* (Oudemans, 1902) | Chamobatidae | 6 (2-9) | 2.80 ± 0.64 | 3.20 ± 1.49 | secondary |
| *Eniochthonius minutissimus* (Berlese, 1903) | Eniochthoniidae | 8 (2-16) | 3.16 ± 1.15 | 5.46 ± 0.88 | secondary |
| *Eupelops tardus* (C.L. Koch, 1835) | Phenopelopidae | 1 (1-1) | 1.90 ± 0.35 | 3.87 ± 0.44 | secondary |
| *Eupelops torulosus* (C.L. Koch, 1839) | Phenopelopidae | 1 (1-1) | 0.99 ± 0.06 | 2.87 ± 0.52 | secondary |
| *Euzetes globulus* (Nicolet, 1855) | Euzetidae | 1 (1-1) | 3.12 ± 1.12 | 2.72 ± 0.26 | secondary |
| *Galumna lanceata* (Oudemans, 1900) | Galumnidae | 2 (1-2) | 2.48 ± 0.32 | 4.65 ± 0.80 | secondary |
| *Nanhermannia cf. coronata* Berlese, 1913 | Nanhermanniidae | 8 (2-14) | 2.98 ± 0.91 | 4.20 ± 0.32 | secondary |
| *Nanhermannia nana* (Nicolet, 1855) | Nanhermanniidae | 3 (3-3) | 3.61 ± 0.76 | 3.12 ± 0.96 | secondary |
| *Nothrus silvestris* Nicolet, 1855 | Nothridae | 3 (1-5) | 3.43 ± 0.67 | 4.67 ± 1.56 | secondary |
| *Ophidiotrichus tectus* (Michael, 1884) | Oribatellidae | 6 (4-8) | 1.65 ± 0.62 | 2.12 ± 1.05 | secondary |
| *Oribatella quadricornuta* Michael, 1880 | Oribatellidae | 2 (2-3) | 2.46 ± 0.40 | 4.64 ± 1.07 | secondary |
| *Oribatula tibialis* (Nicolet, 1855) | Oribatulidae | 3 (1-5) | 2.47 ± 0.35 | 5.68 ± 1.99 | secondary |
| *Scheloribates initialis* (Berlese, 1908) | Scheloribatidae | 4 (3-6) | 2.60 ± 0.60 | 2.99 ± 0.78 | secondary |
| *Achipteria coleoptrata* (Linne, 1758) | Achipteriidae | 6 (1-10) | 2.32 ± 0.93 | 0.23 ± 1.19 | primary |
| *Hermannia gibba* (C.L. Koch, 1839) | Hermanniidae | 2 (2-2) | 0.20 ± 0.41 | -1.52 ± 0.86 | primary |
| *Nothrus palustris* C.L. Koch, 1839 | Nothridae | 1 (1-1) | 1.27 ± 0.31 | 0.22 ± 0.20 | primary |
| *Parachipteria punctata* (Nicolet, 1855) | Achipteriidae | 2 (2-2) | 0.11 ± 0.30 | -0.92 ± 0.78 | primary |
| *Platynothrus peltifer* (C.L. Koch, 1839) | Camissidae | 2 (1-2) | 1.88 ± 0.66 | 0.41 ± 0.38 | primary |
| *Tectocepheus sarekensis* Traegardh, 1910 | Tectocepheidae | 15 (15-15) | 0.75 ± 0.75 | -0.52 ± 0.51 | primary |
| *Tectocepheus velatus* (Michael, 1880) | Tectocepheidae | 11 (5-19) | 1.02 ± 0.53 | 1.59 ± 1.85 | primary |

## Table S3

Cross tabulation summarizing the study design. Frequency of feeding guilds (primary decomposer, secondary decomposer, endophagous and predator Oribatida) in samples of the forest type [Douglas fir (Douglas), Douglas fir/European beech (Douglas/Beech), European beech (Beech), Norway spruce/European beech (Spruce/Beech), Norway spruce (Spruce)].

|  | Douglas | Douglas/Beech | Beech | Spruce/Beech | Spruce |
| --- | --- | --- | --- | --- | --- |
| primary | 12 | 6 | 6 | 4 | 8 |
| secondary | 12 | 18 | 18 | 20 | 12 |
| endophagous | 8 | 4 | 4 | 8 | 12 |
| predator | 8 | 10 | 8 | 4 | 6 |

## Table S4

Linear mixed-effects models on changes in δ^13^C and δ^15^N values between litter and soil (Type III ANOVA). Fixed effects include Depth (litter and soil), Forest type (European beech, Douglas fir, Norway spruce and mixed forests of European beech and Douglas fir and European beech and Norway spruce), Site condition (sandy and loamy sites), and their interactions. Random effects included 40 forest stands. Satterthwaite’s method was used to estimate denominator degrees of freedom (df). Significant P-values are in bold (P ≤ 0.05).


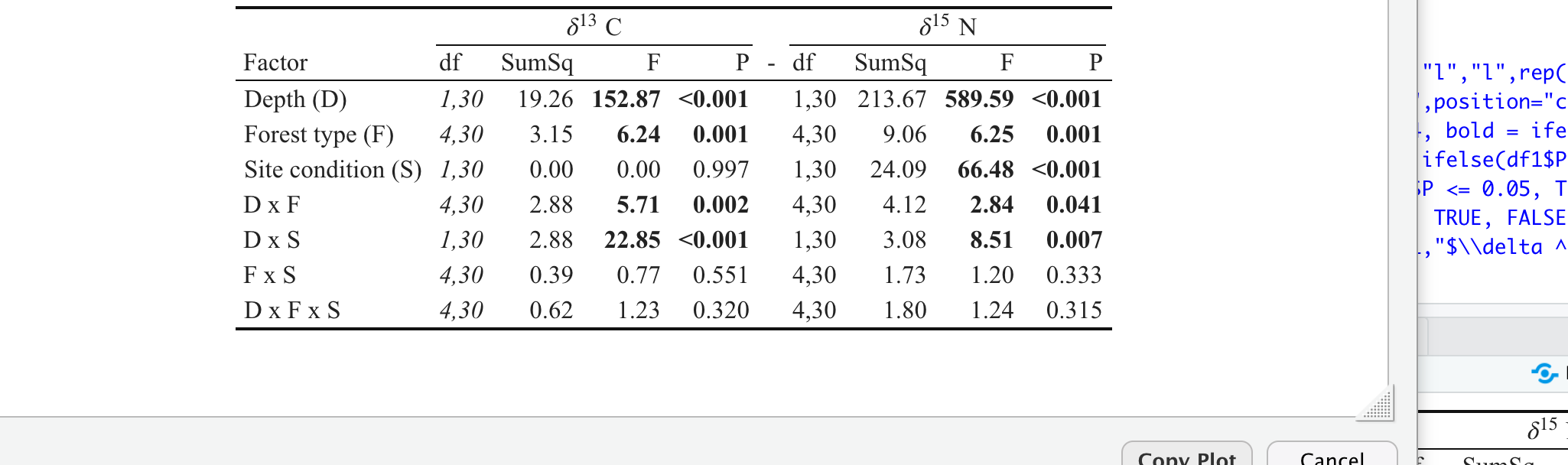


# Supplementary References

﻿Bluhm, C., Scheu, S., Maraun, M., 2016. Temporal fluctuations in oribatid mites indicate that density-independent factors favour parthenogenetic reproduction. Experimental and Applied Acarology 68, 387–407. doi:10.1007/s10493-015-0001-6

Lehmitz, R., Maraun, M., 2016. Small-scale spatial heterogeneity of stable isotopes signatures (δ15N, δ13C) in Sphagnum sp. transfers to all trophic levels in oribatid mites. Soil Biology and Biochemistry 100, 242–251. doi:10.1016/j.soilbio.2016.06.005

Magilton, M., Maraun, M., Emmerson, M., Caruso, T., 2019. Oribatid mites reveal that competition for resources and trophic structure combine to regulate the assembly of diverse soil animal communities. Ecology and Evolution 9, 8320–8330. doi:10.1002/ece3.5409

Maraun, M., Erdmann, G., Fischer, B.M., Pollierer, M.M., Norton, R.A., Schneider, K., Scheu, S., 2011. Stable isotopes revisited: Their use and limits for oribatid mite trophic ecology. Soil Biology and Biochemistry 43, 877–882. doi:10.1016/j.soilbio.2011.01.003

﻿﻿Potapov, A.M., Scheu, S., Tiunov, A. V., 2019. Trophic consistency of supraspecific taxa in below-ground invertebrate communities: Comparison across lineages and taxonomic ranks. Functional Ecology 33, 1172–1183. doi:10.1111/1365-2435.13309/SUPPINFO

Schaefer, I., Caruso, T., 2019. Oribatid mites show that soil food web complexity and close aboveground-belowground linkages emerged in the early Paleozoic. Communications Biology 2, 1–8. doi:10.1038/s42003-019-0628-7

Schneider, K., Migge, S., Norton, R.A., Scheu, S., Langel, R., Reineking, A., Maraun, M., 2004. Trophic niche differentiation in soil microarthropods (Oribatida, Acari): Evidence from stable isotope ratios (15N/14N). Soil Biology and Biochemistry 36, 1769–1774. doi:10.1016/j.soilbio.2004.04.033
